# Critical Evaluation of Strategies for the Production of Blood Coagulation Factors in Plant-Based Systems

**DOI:** 10.3389/fpls.2019.00261

**Published:** 2019-03-07

**Authors:** Oguz Top, Ulrich Geisen, Eva L. Decker, Ralf Reski

**Affiliations:** ^1^Plant Biotechnology, Faculty of Biology, University of Freiburg, Freiburg im Breisgau, Germany; ^2^Spemann Graduate School of Biology and Medicine, University of Freiburg, Freiburg im Breisgau, Germany; ^3^Faculty of Medicine, Institute for Clinical Chemistry and Laboratory Medicine, University of Freiburg, Freiburg im Breisgau, Germany; ^4^Signalling Research Centres BIOSS and CIBSS, University of Freiburg, Freiburg im Breisgau, Germany

**Keywords:** plant molecular farming, biopharmaceuticals, blood coagulation factors, factor II, factor VIII, factor IX, factor XIII

## Abstract

The use of plants as production platforms for pharmaceutical proteins has been on the rise for the past two decades. The first marketed plant-made pharmaceutical, taliglucerase alfa against Gaucher’s disease produced in carrot cells by Pfizer/Protalix Biotherapeutics, was approved by the US Food and Drug Administration (FDA) in 2012. The advantages of plant systems are low cost and highly scalable biomass production compared to the fermentation systems, safety compared with other expression systems, as plant-based systems do not produce endotoxins, and the ability to perform complex eukaryotic post-translational modifications, e.g., *N*-glycosylation that can be further engineered to achieve humanized *N*-glycan structures. Although bleeding disorders affect only a small portion of the world population, costs of clotting factor concentrates impose a high financial burden on patients and healthcare systems. The majority of patients, ∼75% in the case of hemophilia, have no access to an adequate treatment. The necessity of large-scale and less expensive production of human blood coagulation factors, particularly factors associated with rare bleeding disorders, may be an important area for plant-based systems, as coagulation factors do not fit into the industry-favored production models. In this review, we explore previous studies on recombinant production of coagulation Factor II, VIII, IX, and XIII in different plant species. Production of bioactive FII and FIX in plants was not achieved yet due to complex post-translational modifications, including vitamin K-dependent γ-carboxylation and propeptide removal. Although plant-made FVIII and FXIII showed specific activities, there are no follow-up studies like pre-clinical/clinical trials. Significant progress has been achieved in oral delivery of bioencapsulated FVIII and FIX to induce immune tolerance in murine models of hemophilia A and B, resp. Potential strategies to overcome bottlenecks in the production systems are also addressed in this review.

## Blood Coagulation Cascade

**FIGURE 1:**
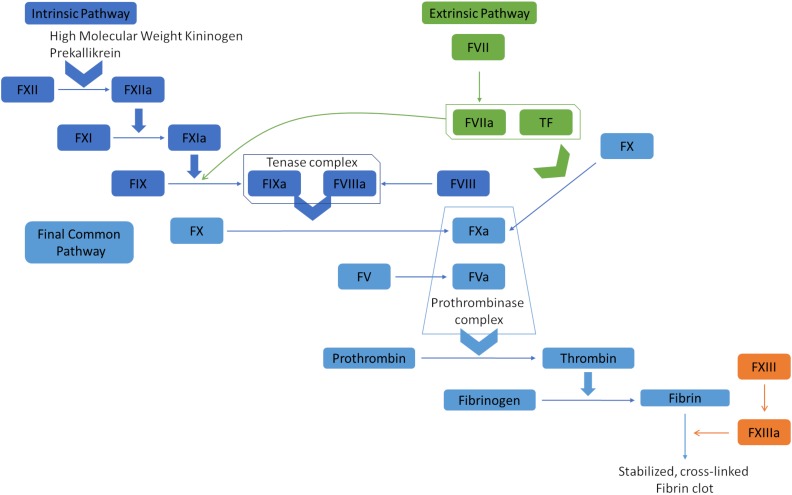
Overview of the blood coagulation cascade (a simplified version). The extrinsic pathway (tissue factor pathway) is initiated with the release of tissue factor (TF), which is a co-factor of Factor VIIa (FVIIa). The formation of TF-FVIIa complex can convert Factor X (FX) to FXa, and Factor IX (FIX) to FIXa. FIXa and FXa propagate the cascade by activating Factor VIII (FVIII) and Factor V (FV). The prothrombinase complex (FVa and FXa) converts prothrombin (Factor II) to thrombin, which processes fibrinogen to fibrin. Fibrin polymerases stabilized and cross-linked fibrin clots. The intrinsic pathway (contact pathway) is initiated by Factor XII (FXII), prekallikrein, and high molecular weight kininogen. FXIIa actiates FXIa, which in turn activates FIX. At that point, the intrinsic and extrinsic pathways converge into the common pathway that leads to clot formation.

Hemostasis is the complex physiological process responsible for stopping bleeding (hemorrhages). It depends on a delicate balance of pro- and anticoagulant forces. The main task of the human blood coagulation system is to prevent excessive blood loss after vascular injury. This is fulfilled by the concerted action of many players including coagulation factors that act in two major pathways, known as extrinsic pathway (tissue factor pathway) and intrinsic pathway (the contact pathway) (Smith et al., 2015). The coagulation factors are mostly serine proteases, except tissue factor (TF), Factor V (FV), Factor VIII (FVIII), and Factor XIII (FXIII). One common feature of the coagulation factors is that they mostly circulate in blood in their inactive zymogen form to maintain homeostasis. They are activated via limited proteolysis upon blood loss from damaged vessels to catalyze the next reaction in the cascade (Smith et al., 2015). The extrinsic pathway is triggered at the site of injury due to the release of TF and hence, is also called tissue factor pathway. TF is a co-factor of Factor VIIa (FVIIa) and the formation of the TF:FVIIa complex catalyzes two downstream reactions: the conversion by proteolysis of Factor X (FX) and Factor IX (FIX) to FXa and FIXa, respectively. On the other hand, the intrinsic pathway begins with Factor XII (FXIIa), high molecular weight kininogen, prekallikrein, and Factor XI (FXI) (Silverberg et al., 1980; Tankersley and Finlayson, 1984; Palta et al., 2014). FXIa and TF:FVIIa complex further activates FIX, which acts with Factor VIII (FVIII) to form the tenase complex to activate FX. FXa propagates the cascade, the final common pathway, by activating FV (Figure 1). Subsequently, prothrombin (Factor II) is activated by the prothrombinase complex (FXa and FVa) and processes fibrinogen into fibrin, which forms a mesh over the wound, activates platelets and forms the blood clot by which the bleeding is stopped (Palta et al., 2014).

Coagulation factor deficiency or dysfunction due to inherited or acquired coagulation disorders impair hemostasis, which can result in life-threatening spontaneous bleeding without an obvious reason. The incidence and severity of disorders are dependent on the amount of clotting factor that is missing in the body. In addition to well-known FVIII and FIX deficiencies, which lead to hemophilia A and B, respectively, there are also other rare bleeding disorders due to deficiencies in Factor I (fibrinogen, FI), FV, FVII, FX, FXI, FXIII, and prothrombin (FII). There are 315,423 people suffering from bleeding disorders [based on data from 116 countries on the Annual Global Survey 2017 by World Federation of Hemophilia (2018)]; 196,706 of them being hemophilia patients, 76,144 von Willebrand disease (VWD), and 42,573 patients with other bleeding disorders. The World Federation of Hemophilia estimates that 400,000 people worldwide are suffering from hemophilia and only 25% receive decent treatment.

Hemophilia A is the most common bleeding disorder caused by defects in the FVIII gene, located on the X chromosome (Mannucci and Tuddenham, 2001). Hemophilia A occurs in 1 in 10,000 live births (1 in 5,000 male) (Ponder, 2011). Patients are grouped into three classes based on the severity of the disease, which is associated with the level of FVIII circulating in the blood. Patients with severe hemophilia A have 1% or less FVIII, moderate hemophilia A patients have between 1 and 5% FVIII and mild hemophilia A patients between 5 and 25% FVIII in circulation (Pool and Shannon, 1965). Mild hemophilia A patients experience reduced hemostasis upon bleeding after major trauma or surgery. However, under severe conditions, patients suffering from more severe hemophilia A do not stop bleeding after a minor trauma or start bleeding spontaneously, especially in joint spaces and soft tissues. Hemophilia B, also known as Christmas disease, is an X chromosome-linked disorder in the FIX gene (Soucie et al., 1998). It occurs in 1 in 30,000 male births and patients can be grouped into three classes, like in Hemophilia A, based on the severity of the disease that corresponds to the level of FIX circulating in the blood (Soucie et al., 1998). Deficiencies in FI, FV, FVII, FX, FXI, FXIII, and prothrombin also cause improper clotting, resulting in patients suffering from similar symptoms like in hemophilia.

There is currently no cure for bleeding disorders and treatment is restricted to protein replacement therapy, either plasma-derived or recombinant products. Therapy can be on-demand treatment or prophylaxis, i.e., the regular supplementation of clotting factor concentrates to keep concentrations over a certain level to prevent bleeding. The average annual per-person medical costs were $85,852 for mild/moderate hemophilia B and $198,733 for severe hemophilia B in the United States (Chen et al., 2017). Even if prophylaxis provides a better life quality, costs increase dramatically. The average medical costs for patients with severe hemophilia A receiving on-demand treatment in the United States were $184,518 p.a., for those receiving prophylaxis $292,525 p.a.. Factor VIII concentrates are the major burden within these costs ($170,037 and $289,172 p.a., respectively) (Chen, 2016). The global market value of recombinant coagulation factors was approximately $8.5 billion in 2017 (Walsh, 2018).

The cloning of the FVIII (Gitschier et al., 1984) and FIX (Choo et al., 1982) genes not only promoted recombinant production of clotting factors but also instigated gene therapy attempts for hemophilia (Mannucci and Tuddenham, 2001). Yet these attempts are still far from offering standardized solutions for patients due to manufacturing and safety concerns of gene therapy vectors and their immunogenic responses triggered in patients (Doshi and Arruda, 2018). Moreover, gene therapy trials are concentrated on the diagnosis of hemophilia but not on other bleeding disorders, mainly due to the fact that these are low incidence diseases, varying from 1 in 500,000 to 1 in 2–3 million (Palla et al., 2015).

Major advantages of plant molecular farming are lower costs and highly scalable biomass production compared to typical fermentation systems (Edgue et al., 2017; Margolin et al., 2018). Plant-based systems are generally safer because they do not bear the risks of product contaminations with endotoxins, infectious viruses, and prions (Reski et al., 2015). Plants can also perform complex post-translational modifications, e.g., Asn-linked (*N*-) glycosylation, that can be further engineered to achieve humanized *N*-glycan structures (Castilho et al., 2010; Castilho and Steinkellner, 2012; Decker and Reski, 2012). Attempts on the prevention of plant-specific *O*-glycosylation and the establishment of *de novo* humanized *O*-glycosylation, despite current limitations, show another advantage of plant systems (Schoberer and Strasser, 2018). The necessity of large-scale and less expensive production of human blood coagulation factors, particularly factors associated with rare bleeding disorders, may be an important area for plant-based systems, as coagulation factors do not fit into the industry-favored production models.

In this review, we summarize the previous studies on recombinant production of FII, FVIII, FIX, and FXIII in plant systems. We also discuss the current challenges and provide possible solutions to overcome bottlenecks.

## Factor VIII

**FIGURE 2:**
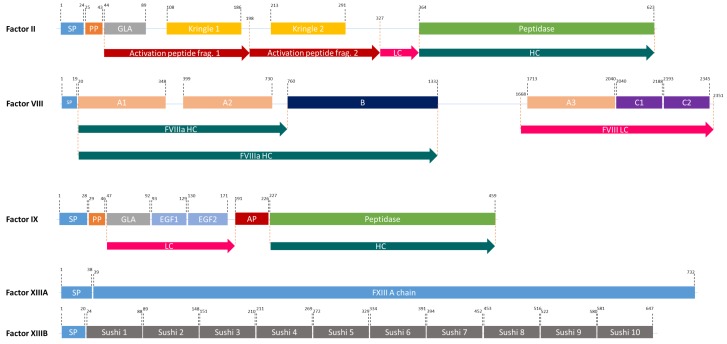
The domain structures of FII, FVIII, FIX, and FXIII (a heterodimer of 2 FXIIIA, and 2FXIIIB). SP, Signal peptide; PP, Propeptide; GLA, Domain containing γ-carboxyglutamic acid residues; LC, Light chain; HC, Heavy chain; EGF, Epidermal growth factor like domain; AP, Activation peptide.

The FVIII gene spans a genomic region of 186 kb, which contains 26 exons and 25 introns (Oldenburg et al., 2004) and is transcribed into 9 kb long mRNA, that encodes a single-chain protein with 2,322 amino acids (Gitschier et al., 1984). FVIII consists of six major domains (A1-A2-B-A3-C1-C2, Figure 2) and is mainly produced by liver sinusoidal endothelial cells (Do et al., 1999). It circulates in an inactive form bound to von Willebrand factor (vWF) until injury (Fay, 2006) and is activated by the cleavage and release of the B domain, which yields the light chain of 80 kDa and the heavy chain (A1-A2 domains) of variable size (90–200 kDa) (A3-C1-C2 domains) (Thorelli et al., 1998). The deletion of the B-domain increases the product yield but does not affect *in vitro* and *in vivo* activity (Kessler et al., 2005).

Factor VIII is heavily glycosylated, by both, *N*- and *O*-glycosylation, and bears sulphated tyrosine residues (Vehar et al., 1984; Kaufman and Pipe, 1999). *N*-glycosylation was validated for 19 of 25 putative sites in plasma-derived FVIII (pdFVIII) (Canis et al., 2018). The MALDI-MS profiling of pdFVIII revealed that high mannose and complex-type *N*-glycans preponderated: 16% of the population with high mannose, 67% with terminal sialic acid (both α2,3 and α2,6 linkages), and 10% with AB0(H) blood group antigens. Nearly 40% loss of activity after deglycosylation of FVIII illustrated that glycosylation is important for biological activity (Kosloski et al., 2009). Furthermore, terminal sialic acids increase half-life of glycoproteins in plasma by preventing exposure of *N*-acetylgalactosamine, a particular form of *O*-glycosylation, or galactose which are recognized by the asialoglycoprotein receptor in the liver and subsequently cleared from circulation (Ashwell and Harford, 1982). Due to the importance of complex post-translational modifications for FVIII activity, prokaryotic and yeast expression systems are no suitable production hosts (Pipe, 2008).

In addition to the decoration with *N*-linked oligosaccharides to the corresponding sites and the initial processing within the ER, FVIII binds to chaperone proteins, calnexin (CNX), calreticulin (CRT), or binding immunoglobulin protein (BiP), for proper folding (Kaufman, 1998; Molinari et al., 2003; Oda et al., 2003). After the quality control by CNX/CRT, FVIII detaches from these chaperones and can proceed in the secretory pathway (Kaufman, 1998). Here, *N*-linked oligosaccharides are processed and *O*-glycosylation and sulphation of tyrosine residues occur in the Golgi apparatus (Thim et al., 2010; Orlova et al., 2013). On the other hand, FVIII forms a stable complex with BiP, an additional key component in the unfolded protein response (UPR) pathway, in the ER lumen and this interaction is the limiting step in FVIII secretion in Chinese Hamster Ovary (CHO) cells (Dorner et al., 1988; Dorner and Kaufman, 1994). The overexpression and incorrect folding of FVIII lead to the upregulation of BiP and hence the activation of UPR (Soukharev et al., 2002). Deletion of the B-domain, which corresponds to 38% of the sequence and contains the majority of *N*-linked oligosaccharides, and putative BiP binding site on FVIII resulted in increased FVIII secretion (Dorner et al., 1987; Saenko et al., 2003). On the other hand, mutating BiP in CHO cell lines expressing B-domain-deleted FVIII adversely affected FVIII secretion (Morris et al., 1997). These aspects have not been investigated thoroughly in plant systems.

Additionally, there are factors other than BiP binding site and FVIII B-domain limiting FVIII expression in human cell lines that should be taken into account to achieve efficient expression in plant-based systems. It was shown that the 1.2-kb long cDNA coding for the FVIII A2 domain had detrimental effects on RNA accumulation in human cell lines (Lynch et al., 1993). Furthermore, FVIII transcription could be inhibited due to the presence of a 305-bp transcriptional silencer originating from exons 9–11 (Hoeben et al., 1995). Although the majority of the transcriptional regulation mechanisms are shared between plants and animals, there are subtle differences (Macrae and Long, 2012) and one might test whether these limiting factors in human cell lines will also be limiting in plants or plant-based systems. Another limiting factor in human cell lines is that heavy and light chains are degraded in cell culture media quickly. This problem was overcome by the addition of von Willebrand factor (vWF) into cell culture medium and/or co-expression of vWF together with FVIII, as vWF assists FVIII secretion and association of heavy and light chains (Fay, 1988; Saenko et al., 1999). Accumulation of FVIII in plant apoplasts might prevent degradation of recombinant FVIII and plant-based systems may be advantageous for FVIII production.

The first recombinant FVIII, Recombinate^®^, was launched in 1992 by Genetics Institute and Baxter Healthcare Corporation. With the research on FVIII over the years, there are now more than ten recombinant FVIII concentrates on the market compared to over 40 plasma-derived FVIII concentrates [Annual Global Survey 2017 by World Federation of Hemophilia (2018)]. These are produced in CHO cells (seven products), human embryonic kidney (HEK-293) cells (two products), and baby hamster kidney (BHK) cells (two products) (Swiech et al., 2017). Although production is restricted to these three cell types, the characteristics of rFVIII, purification, and the use of animal-derived proteins as stabilizer do differ. Due to the potential risk of exposure to transmissible agents, human albumin was no longer used in the majority of products on the market except Recombinate^®^ and Kogenate^®^ (Swiech et al., 2017). In addition to full-length rFVIII products (Recombinate^®^, Kogenate^®^, Kogenate^®^ FS, Advate^®^, Adynovate^®^, Kovaltry^®^, Jivi^®^), there are also B-domain truncated (Novoeight^®^ and Afstyla^®^) and B-domain deleted (Refacto, Refacto^®^ AF^®^, Eloctate^®^, and Nuwiq^®^) rFVIII products on the market (Swiech et al., 2017). Moreover, to achieve longer half-life and decrease FVIII administration to the patients, FVIII was PEGylated (Adynovate^®^ and Jivi^®^) and even fused to the IgG1-FC (Eloctate^®^) (Swiech et al., 2017). Recently, Hemlimbra^®^ (emicizumab-kxwh), a humanized bispecific monoclonal antibody that restores FVIII function by bridging FIXa and FXa, has been approved by FDA for hemophilia A without FVIII inhibitors (Oldenburg et al., 2017; Scott and Kim, 2018).

The expression of full-length FVIII was achieved in transgenic tobacco lines (Hooker et al., 1999). FVIII levels reached up to 0.002% of soluble leaf protein as confirmed by Western blot analysis. The activity of tobacco-made FVIII in chromogenic assays was 14.85 IU/mg soluble leaf protein (Hooker et al., 1999). Typical levels of FVIII production in CHO cell lines are 0.5–2 IU/ml culture medium (Orlova et al., 2017). Full-length FVIII, as well as B-domain deleted FVIII, and A2-domain exchanged FVIII (human A2 sequence was replaced with porcine due to the adverse effect of the human A2-domain on RNA accumulation) were produced in tobacco protoplasts, whole plants and in calli (Hooker et al., 1999). In addition to the production in tobacco, FVIII was produced in potato (Hooker et al., 1999). In 2014, FVIII domains were produced in tobacco chloroplasts and bioencapsulated in plant cells for oral delivery for patients suffering from hemophilia A with a FVIII inhibitor. FVIII inhibitor development is a helper-T-cell dependent response upon treatment with FVIII concentrates and causes increased morbidity and mortality (Verma et al., 2010; Sherman et al., 2014). Inhibitors, which occur in 20–30% of hemophilia A patients, can be eliminated with immune tolerance-induction (ITI) therapy in which the immune system is trained to tolerate FVIII concentrate by the repeated and frequent administration of FVIII over several months, or sometimes years (Schep et al., 2018). Orally administered tobacco cells expressing FVIII domains in a murine model of hemophilia A suppressed the inhibitor formation by induction of specific populations of regulatory T cells (CD4^+^CD25^+^ and CD4^+^CD25^-^, resp.; Sherman et al., 2014). Recently, full-length FVIII produced in lettuce chloroplasts reached levels up to 852 μg/g in lyophilized plant cells and its oral delivery within lettuce cells suppressed the inhibitor formation in a hemophilia A mouse model (Kwon et al., 2018). Although it was shown that tobacco and lettuce chloroplast-derived foreign proteins were folded properly (Boyhan and Daniell, 2011; Zhang et al., 2017), *N*-glycosylation of proteins in chloroplasts is not possible. Still, the production of FVIII in different plant systems, and especially its expression and bioencapsulation in tobacco and/or lettuce cells for oral delivery to lower inhibitor formation, has a promising future with additional benefits. These benefits are elimination of expensive cell growth and purification costs as well as the suitability and oral delivery of freeze-dried plant cells with proteins for long-term storage without adverse effects on folding and function (Kwon et al., 2018).

## Factor IX

FIX is one of the serine proteases in the blood coagulation cascade and its deficiency causes hemophilia B. FIX is produced in the liver and it is a smaller and less complex protein compared to FVIII (Swiech et al., 2017). However, it undergoes complex post-translational modifications (PTMs) including γ-carboxylation by γ-glutamyl carboxylase (GGCX) in the ER and a proteolytic processing by paired basic amino acid cleaving enzyme (PACE, also called furin) in the Golgi apparatus (Pipe, 2008; Figure 2). Human pdFIX has two sites for *N*-glycosylation (Asp-157 and Asp-164 located in the activation peptide) and four sites for *O*-glycosylation; Ser-53 and Ser-61 which are fully glycosylated and Thr-159 and Thr-169 which are only partially glycosylated (Agarwala et al., 1994; Kaufman, 1998). FIX is secreted into the bloodstream after these extensive PTMs in an inactive zymogen form (∼57 kDa). In the case of bleeding, it is activated by FXIa or FVIIa (Orlova et al., 2012).

Although there are promising attempts to cure hemophilia B via gene therapy (Pfizer announced the initiation of the phase III program for investigational hemophilia B gene therapy, Identifier NCT03587116, in July 2018.), current treatments are restricted to protein replacement therapies, as in hemophilia A. BeneFIX^®^, the first recombinant FIX product, was introduced to the market by Pfizer in 1997, and for a long time it was the only recombinant product on the market (Swiech et al., 2017). Currently, there are additional recombinant products on the market (Rixubis^®^, Alprolix^®^, Ixinity^®^, Idelvion^®^, and Rebinyn^®^). Four of these products are produced in CHO cells and one in HEK-293 cells. Ixinity is the only product with Thr-148 polymorphism, while the rest have Ala-148 (Swiech et al., 2017). Due to the concerns about the half-life of FIX concentrates, several different strategies were employed: three products with improved half-life, Alprolix (FIX-IgG1 Fc fusion), Idelvion (FIX-Albumin fusion) and Rebinyn (PEGylated FIX), were approved by the FDA in 2014, 2016, and 2017, respectively (Powell et al., 2013; Santagostino, 2016; Graf, 2018).

FIX, FVII, FX, prothrombin (FII), protein C, protein S, and osteocalcin are vitamin K-dependent (VKD) proteins and they all have γ-carboxyglutamate (Gla) by the addition of a carboxyl group to a glutamic acid residue. During this process, vitamin K hydroquinone is oxidized to vitamin K 2,3 epoxide and CO_2_ is added to Glu. This reaction is catalyzed by GGCX, an integral membrane protein located in the ER of hepatocytes (Presnell and Stafford, 2002). GGCX recognizes propeptide regions and performs all modifications at once. In its native form, FIX contains 12 Gla residues in the so-called Gla domain; the first 10 Gla residues are conserved in all VKD proteins, whereas the last two are unique to FIX (Gillis et al., 2008). The Gla domain is crucial for the interaction with phospholipid surfaces and consequently for FIX activity (Pipe, 2008). After γ-carboxylation in the ER, FIX is further processed by PACE in the Golgi apparatus. The removal of the propeptide by PACE influences the formation of Ca^2+^-induced secondary and tertiary structures of the Gla domain, thus it is required for normal function of FIX (Pipe, 2008). γ-carboxylation by GGCX and propeptide removal by PACE/furin do not occur *in planta.* Thus, expression of FIX, GGCX, and PACE in plant-based systems is required for a production of bioactive FIX in plants. γ-carboxylation and propeptide removal are rate-limiting steps in FIX production: The overexpression of FIX in CHO cells resulted in 180 μg FIX/ml of culture supernatant but only 0.8% of it was fully carboxylated (Kaufman et al., 1986). When compared to other VKD proteins, one can speculate that FIX might not be the best substrate for GGCX: *In vitro* γ-carboxylation analyses showed that the K_m_ of FIX was several thousand-fold lower than other VKD proteins that have FLEEL or FLEEV peptides, which affects GGCX binding (Wu et al., 1990). Hence, to achieve increased γ-carboxylation in FIX, the propeptide of FIX might be replaced with a better one from other VKD proteins.

The attempts to produce bioactive FIX in plants are challenging due to the absence of GGCX and PACE and the introduction of these two genes alone might not guarantee the functionality of plant-made FIX. As described previously, GGCX converts Glu to Gla by reducing vitamin K hydroquinone to vitamin K epoxide. Plants are the main source of vitamin K and animals are dependent on plants (Shearer and Newman, 2008). Most likely due to the limited availability of vitamin K in animals, vitamin K epoxide has to be converted first to vitamin K quinone and then to vitamin K hydroquinone which can be used in γ-carboxylation (Stafford, 2005). These reactions are catalyzed by the vitamin K epoxide reductase complex (VKORC) subunit 1, which is also absent in plants. Another challenge is that FIX has to be γ-carboxylated in the ER but vitamin K is synthesized from shikimate by nine consecutive reactions taking place mainly in chloroplasts and partially in peroxisomes, and the final product, vitamin K phylloquinone, is located in the chloroplast (Reumann, 2013). Even though it was proposed that chloroplasts are metabolically coupled to the ER (Bobik and Burch-Smith, 2015), to our knowledge there is no study on the presence of vitamin K in the ER of plants. Therefore, to achieve at least a limited amount of vitamin K in the ER, one can suggest feeding the plants with vitamin K and introducing VKORC1 to ensure enough vitamin K hydroquinone is generated, which GGCX needs during the γ-carboxylation process. Moreover PACE, the propeptide removal enzyme, has its own propeptide and undergoes a complex self-activation process in animals: The propeptide in PACE first undergoes Ca^2+^ autoproteolysis in the ER and second Ca^2+^ and acidic pH-dependent autoproteolysis in the *trans-*Golgi network. After these two cleavages, the propeptide is completely removed from PACE and PACE is converted into the active form (Anderson et al., 1997, 2002). The attempts to produce PACE without the propeptide resulted in non-functional PACE due to the fact that the propeptide regulates folding of the protein (Ware et al., 1989; Bristol et al., 1993). Consequently, expression of full-length PACE/furin and its Ca^2+^ and acidic pH-dependent autoproteolysis are also important for plant-based systems. During maturation of FIX, GGCX has to act before PACE because if PACE acts first, GGCX cannot bind and modify FIX. Thus, the intracellular localization of GGCX and PACE, if introduced into plant-based systems, has to be guaranteed. There is no report on the introduction of GGCX in plants yet, but one study reported the successful introduction of PACE/furin in *Nicotiana benthamiana* and confirmed its activity on transforming growth factor-β1 (Wilbers et al., 2016). With this report (Wilbers et al., 2016), it can be anticipated that PACE can also activate itself by Ca^2+^ and acidic pH-dependent autoproteolysis in plants, despite the differences between plant and animal organelles’ pH (Shen et al., 2013).

There have been several attempts to produce FIX in plant-based systems. The first study aimed to accumulate FIX in tomato fruits and reached up to 0.01584 mg FIX/g fresh weight fruit (Zhang et al., 2007). In the second study, FIX was introduced into soybean and the highest FIX levels were 800 mg/kg of soybean seeds (Cunha et al., 2011). However, plant-made FIX proteins failed to show any activity due to the fact that only FIX (without GGCX, PACE, and VKORC1) was expressed. In another study, as in FVIII bioencapsulated in lettuce cells (Kwon et al., 2018), FIX was produced in lettuce chloroplasts and oral delivery of bioencapsulated FIX in lettuce cells to the hemophilia B murine model suppressed inhibitor formation (Su et al., 2015).

It is still possible to achieve *in vitro* γ-carboxylation (Hubbard et al., 1989), but it has several disadvantages. Isolation of liver microsomes, purification of GGCX from microsomes, control and completeness of the *in vitro* γ-carboxylation assay, heterogeneity of end products and necessity for further purification and the associated costs make *in vitro* γ-carboxylation inapplicable. Attempts to produce bioactive FIX in plant-based systems are currently not possible due to complex post-translational modifications. Once current challenges will have been overcome, plant-based systems might become a good alternative production host for this blood coagulation factor also.

## Factor XIII

FXIII is a transglutaminase that stabilizes the fibrin clot by crosslinking fibrin monomers and protecting the clot from fibrinolytic degradation (Kaufman and Pipe, 1999; Lovejoy et al., 2006). It circulates in the plasma as a tetramer of two A and B subunits (Komáromi et al., 2011; Figure 2). FXIII-B subunits are present in plasma freely in excess amounts (Komáromi et al., 2011). FXIII deficiency causes a rare bleeding disorder, affecting one in 1–5 million people, due mainly to mutations in the FXIII-A subunit (Karimi et al., 2009). Patients suffering from this deficiency require life-long protein replacement therapy (Komáromi et al., 2011).

The A subunit of FXIII has no signal sequence, no *N*-linked glycosylation, and no sulfides (Mosher, 2014). Thus, in this case yeast systems can be preferred as production host over plants and animals. Hence, the recombinant product (rFXIII-A_2_) on the market is produced in *Saccharomyces cerevisiae* by Novo Nordisk. The recent trial with patients showed that yeast-made rFXIII-A_2_ prevented bleeding and did not cause the formation of any non-neutralizing or neutralizing antibodies in patients (Carcao et al., 2018). The A subunit was also successfully expressed in tobacco cell suspensions (NT-1) and in tobacco plants (Gao et al., 2004). Tobacco-made FXIII-A reached up to 1.8% of the total extracted soluble leaf protein and showed FXIII-specific activity of 258 U/g of soluble protein, while human plasma-derived FXIII has an activity of 50.2 U/mg of soluble protein (Gao et al., 2004). Moreover, like human plasma-derived FXIII, it crosslinked human fibrin and produced dimers and multimers (Gao et al., 2004). To our knowledge, there was no follow-up study.

## Prothrombin

Prothrombin (FII), a member of vitamin-K dependent proteins, is a 72 kDa protein composed of 579 amino acids (Mann et al., 2003). It comprises four major domains: a Gla domain, two kringle domains and a catalytic domain (Soriano-Garcia et al., 1992). Like FIX, it is modified by GGCX and PACE. It has four cleavage sites: R^155^, R^271^, R^284^, and R^320^ (Suttie and Jackson, 1977; Figure 2). Two of these (R^284^ and R^320^), are cleaved by FXa and the other two are thrombin-specific auto-proteolytic sites. Depending on the cleavages, prethrombin 1, prethrombin 2, meizothrombin, fragment 1, and fragment 1.2 are generated from prothrombin (Galli and Barbui, 1999). The most important product after possible cleavages is prethrombin 2, which is an inactive FII intermediate that is further cleaved by FXa to form active thrombin (Boskovic et al., 1990). This active protein can be used in any surgical procedure to control excessive intra-operative bleedings (Croxtall and Scott, 2009). Therefore, rather than producing full-length FII, attempts concentrated on the production of functional isoforms. In 2008, Recothrom^®^, rThrombin produced in CHO cells, by Zymogenetics received United States market approval (Ratner, 2008).

Expression of prothrombin and prethrombin 2 is possible in tobacco plants but unfortunately, the specific activity of tobacco-made prethrombin 2 was not determined (Hooker et al., 1999). Although GGCX and PACE activities are required for the maturation of prothrombin, in the active thrombin, there is no Gla residue. Moreover, there is only one *N-*linked glycosylation site in the active form. Due to these reasons, plants can still be an alternative platform in the production of prothrombin, even without expression of GGCX and PACE, or prethrombin 2, as long as plant-made prothrombin or prethrombin 2 can be activated by factor Xa. However, this suggestion has to be tested and costs, especially for *in vitro* activation, have to be compared with other production hosts, especially human cell lines.

## Outlook

Despite an increasing interest in using plants as alternative expression hosts, studies on plant-made blood clotting factors revealed that plant-based systems are no alternative platform as long as current problems are not solved. Yields of recombinant plant-made blood clotting factors can be enhanced by modulating many factors such as promoter and terminator activities, codon optimization of the transgene, matrix attachment regions, mRNA stability, translational efficiency, vector size, viral systems, and silencing suppressors. Production of bioactive vitamin K-dependent coagulation factors (FII, FVII, FIX, and FX) has not been achieved in plant-based systems yet. Vitamin K supplementation and expression of FIX, GGCX, PACE, and VKORC1, which have not been reported yet, can overcome current bottlenecks. Improvements in plant-made pharmaceuticals’ quality and quantity are as important as maximizing the product yield (Buyel et al., 2015). Optimization of downstream processes and associated costs have not been addressed in previous plant-made clotting factor studies. To make plant-based systems competitive alternatives, upstream as well as downstream processes have to be performed in an advanced and cost-effective manner compared to traditional expression systems.

Glycosylation can affect structure and function of the protein and differences in plant and human glycosylation can induce immune responses in patients (Kosloski et al., 2009). Although the principles of protein Asn-linked (*N*) glycosylation and *N*-glycan core structures are identical between plants and humans, there are differences in specific proximal and terminal sugar residues within the glycan structures. Plant-specific α1,3-fucose and β1,2-xylose residues can be immunoreactive in humans, hence it is desirable to produce proteins with humanized glycosylation (Bardor et al., 2003; Gomord et al., 2010; Decker et al., 2014; Shaaltiel and Tekoah, 2016). For this purpose, the first step was to knock out plant-specific sugar residues by glyco-engineering. This was achieved for the first time by knocking out *FucT* and *XylT* genes in *Arabidopsis thaliana* (Strasser et al., 2004) and in *Physcomitrella patens* (Koprivova et al., 2004). Due to the high homologous recombination rates in the moss *P. patens*, *FucT* and *XylT* genes have been knocked out and human β-1,4-galactosyltransferase was stably introduced into the moss genome a year later (Huether et al., 2005). With the advent of CRISPR/Cas9 technology, six genes responsible for xylose and fucose residues were knocked out in *Nicotiana tabacum* BY-2 suspension cells (Hanania et al., 2017; Mercx et al., 2017) and in *N. benthamiana* (Jansing et al., 2018) more recently. Moreover, overexpression of mammalian sialic acid biosynthesis pathway genes in *N. benthamiana* enabled *in-vivo* sialylation (Castilho et al., 2010; Castilho and Steinkellner, 2012).

Unlike *N*-glycosylation, which is partially conserved between all eukaryotes, plant *O*-glycosylation is fundamentally different from the typical human mucin-type *O*-glycosylation. In mucins, *O*-glycans are incorporated via an *N-*acetylgalactosamine to the hydroxyl side of serine or threonine residues in the Golgi apparatus. Neither these *O*-glycans nor the glycosyltransferases for the human mucin-type *O*-glycosylation are present in plants (Taylor et al., 2012; Tryfona et al., 2012; Saito et al., 2014). Although a single Gal attachment to Ser residues on specific proteins is observed in plants, mainly arabinose chains and complex arabinogalactans are attached to 4-*trans-*hydroxyproline (Hyp), whereas no modification on Hyp is observed in mammals (Strasser, 2013). Hence, the elimination of non-human prolyl-hydroxylation, which might potentially be the cause of immunogenic response in patients, can be a safe strategy to avoid adverse effects of plant-made pharmaceuticals. Addition of the prolyl 4-hydroxylase inhibitor 2,2-dipyridyl to *N. tabacum* Bright Yellow-2 suspension cultures abolished proline hydroxylation and arabinosylation (Yang et al., 2012). Moreover, targeted knockout of *P4H1*, the gene for non-human prolyl-hydroxylation of human erythropoietin recombinantly produced in *P. patens*, eliminated the attachment of plant-specific *O*-glycosylation (Parsons et al., 2013). The success of humanized *N*-glycosylation in plants (Castilho et al., 2010, 2013; Castilho and Steinkellner, 2012) inspires studies on *de novo* *N*-acetylgalactosamine (GalNAc) type *O*-glycosylation in plants. The attachment of a single GalNAc residue was achieved for the first time with the transient expression of UDP-GlcNAc 4-epimerase (*Yersinia enterocolitica*), UDPGlcNAc/UDP-GalNAc (*Caenorhabditis elegans*), and human GalNAc-T2 in *N. benthamiana* (Daskalova et al., 2010). Subsequently, transgenic *A. thaliana* and tobacco BY2 cells were generated (Yang et al., 2012). Despite the significant progress, there are still limitations to achieve efficient humanized *O*-glycosylation in plant-made pharmaceuticals, such as heterogeneous plant-produced *O*-glycan structures, formation of core 2, 3, and 4 structures, and optimization of sub-Golgi targeting of mammalian glycosyltransferases in plants (Strasser, 2013). Taken together, several bioengineering challenges have to be addressed to really evaluate the competitiveness of plant-made clotting factors and plant-based systems in this field of biopharmaceuticals.

## Conclusion

Plant-based systems are highly scalable, cost-effective, GMP-compliant, and safer than animal systems. They are successful in humanized *N*-glycosylation, and promising progress in mucin type *O*-glycans revealed that they are becoming competing alternatives to mammalian cells. Although studies summarized in this review show that plant-based systems are able to produce some bioactive blood coagulation factors, to the best of our knowledge, there were no further studies to fully characterize the potential of plant-made clotting factors in pre-clinical and clinical trials. The production of functional FIX and prothrombin is still challenging, as γ-carboxylation was not yet achieved in plant-based systems. Approaches aiming to acquire plant-made coagulation factors with enhanced pharmacological properties by engineering the glycosylation pathway as well as optimizing plant-based systems to meet the demands of industry norms will reveal the potential of plant systems.

## Author Contributions

OT, ED, and RR wrote the manuscript. UG contributed critical comments to the draft. All authors have read and approved the final manuscript.

## Conflict of Interest Statement

The authors declare that the research was conducted in the absence of any commercial or financial relationships that could be construed as a potential conflict of interest.
